# Acute Coronary Syndrome Manifesting as an Adverse Effect of All-*trans*-Retinoic Acid in Acute Promyelocytic Leukemia: A Case Report with Review of the Literature and a Spotlight on Management

**DOI:** 10.1155/2016/2829142

**Published:** 2016-02-11

**Authors:** K. Govind Babu, K. N. Lokesh, M. C. Suresh Babu, Gita R. Bhat

**Affiliations:** Department of Medical Oncology, Kidwai Memorial Institute of Oncology, Dr. M. H. Marigowda Road, Hombegowda Nagar, Bengaluru, Karnataka 560030, India

## Abstract

*Background.* Acute promyelocytic leukemia is characterized by t(15;17). This leads to the formation of PML/RAR*α* which blocks the differentiation of blasts at the stage of promyelocytes. This is reversed by all-*trans*-retinoic acid (ATRA), a vitamin A derivative. Acute myocardial ischemia is a rare side effect of ATRA.* Case Report.* We report a case of acute coronary syndrome manifesting as an adverse effect of ATRA in a lady with APL who had no other risk factors for cardiovascular disease.* Conclusions.* We emphasize the need for high index of suspicion for the diagnosis of this entity. In the light of this case, the rare instances of ATRA associated acute myocardial ischemia recorded in the literature and the options available for treatment of acute promyelocytic leukemia sans ATRA have been reviewed.

## 1. Background

Acute promyelocytic leukemia (APL) is a subtype of acute myeloid leukemia characterized by reciprocal translocations between the long arms of chromosomes 15 and 17 [t(15;17)]. This leads to the fusion of the retinoic acid receptor and the promyelocytic leukemia (PML) genes, called PML/RAR*α*. PML/RAR*α* gene product is a transcription repressor. It blocks the differentiation of APL blasts at the stage of promyelocytes. Pharmacologic doses of all-*trans*-retinoic acid (ATRA) reverse this blockage and induce remission. Thus, ATRA has revolutionized the treatment of APL [1–3]. Acute myocardial ischemia is an infrequent adverse effect of ATRA. There are only few reported cases describing this complication [4–8]. We report a case of acute coronary syndrome manifesting as an adverse effect of ATRA in a lady with APL. To our knowledge, ours was the youngest patient to be diagnosed with ATRA induced acute myocardial ischemia. She had neither comorbidities nor APL-DS. We have also described the use of single-agent arsenic trioxide as a valid treatment option in this clinical scenario.

## 2. Case Report

A 42-year-old lady presented to our institute with a 10-day history of menorrhagia, multiple bruises, and fatigue. She had multiple ecchymotic patches, petechiae, and bilateral retinal hemorrhages. At admission, her hemoglobin was 10.4 g/dL, WBC count was 5,900/mm^3^, and platelet count was 31,000/mm^3^. Peripheral smear revealed numerous promyelocytes, some with Auer rods. Flow cytometry was done on the bone marrow aspirate. Gated cells in the blast region were positive for CD13, CD33, MPO, and CD117 and negative for HLA-DR and CD 34. Cytogenetic study by Giemsa banding carried out on the cells from the bone marrow aspirate showed 46, XX t(15;17) karyotype. PML/RAR*α* translocation was not detected by quantitative minor groove binder real time PCR assay. This assay can detect bcr1 (long form) and bcr3 (short form) of PML/RAR*α*. However, the qualitative assay indicated the presence of variant form of PML/RAR*α* transcript, bcr2, with the PML breakpoint at exon 6. Serum fibrinogen level was 247 mg/dL (range: 180–350 mg/dL), and prothrombin time (international normalized ratio) (PT INR) was prolonged; there was no clot formation for >120 seconds. Activated partial thromboplastin time (aPTT) was prolonged (>50 seconds). These findings were compatible with the diagnosis of APL, intermediate risk with variant form of PML-RAR*α*, and disseminated intravascular coagulation (DIC).

As per institution protocol, treatment with ATRA 45 mg/m^2^ body surface area/day, in two divided doses, orally, and arsenic trioxide (ATO) 0.15 mg/kg/day as an intravenous infusion in 100 mL of normal saline once daily, platelet transfusions, and fresh frozen plasma transfusions was started immediately. After nine days of therapy, her coagulation profile returned to normal. There was no evidence of APL differentiation syndrome (APL-DS). Her coagulation profile during the course of treatment has been summarized in Table 1.

**Figure 1:**
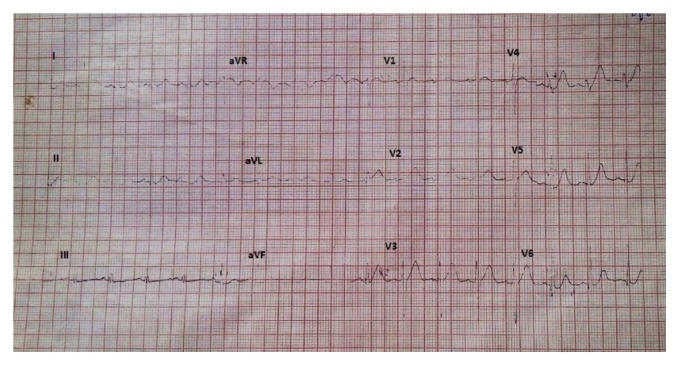
Baseline electrocardiogram.

Ten days after commencement of treatment with ATRA, she had sudden onset of retrosternal pain and dyspnea. Her pulse rate was 100 beats per minute. Blood pressure was 100/70 mmHg. Rales were heard in bilateral lung bases, suggestive of left ventricular failure. She never had chest pain or exertional dyspnea in the past and had no specific risk factors for ischemic heart disease such as diabetes, hypertension, obesity, or dyslipidemia. She did not smoke and was not on oral contraceptives nor was there family history of myocardial infarction. No other drugs had been administered between admission and the time of the event. Electrocardiogram (Figure 1) and multigated acquisition mode study (MUGA study) carried out prior to initiation of therapy were normal. Left ventricular ejection fraction (LVEF) was 51% (range 50–70%).

**Figure 2:**
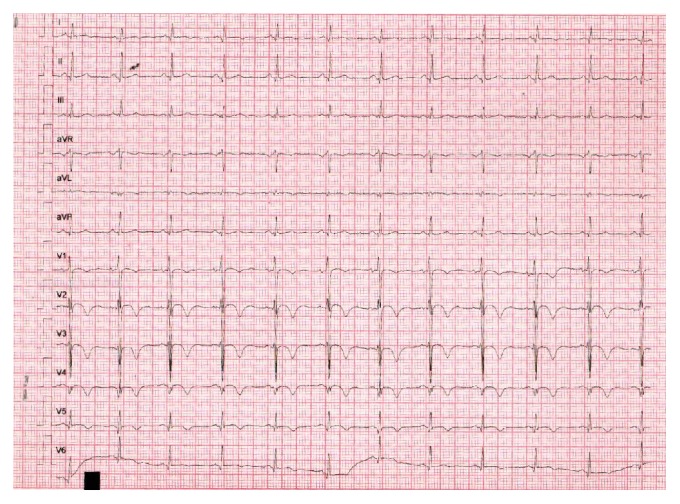
Electrocardiogram obtained during the episode of chest pain. It shows inversion of T wave in leads I, aVL, and V2–V6. There is poor progression of the R wave.

We sought an emergency cardiology opinion. Electrocardiogram showed T wave inversions in leads I, aVL, and V2–V6 and poor progression of R wave (Figure 2). Troponin T was 0.734 ng/mL (normal: <0.1 ng/mL). Creatine kinase-MB isoenzyme was 34 U/L (range: 0–25 U/L). Two-dimensional echocardiography revealed thinning and hypokinesia of midanteroseptal, apicoseptal, and apicoanterior segments of the left ventricle. LVEF was 41% (range: 50–70%). Hemoglobin was 8.8 g/dL, total leukocyte count was 8,100/mm^3^, and platelet count was 40,000/mm^3^. She was prescribed metoprolol (25 mg/day), ramipril (2.5 mg/day), glyceryl trinitrate (2.6 mg/day), and spironolactone (25 mg/day). Due to thrombocytopenia she did not receive antiplatelet therapy and invasive coronary angiography was deferred. Three days later, she was asymptomatic and was discharged from the cardiology ward.

We were faced with the challenge of the treatment of APL as we had limited options. The Naranjo Adverse Drug Reaction (ADR) Probability Scale was used [9]. Our case was assigned to the category of “probable” ADR to ATRA based on the score of 7 (Table 2). She was not rechallenged with ATRA in order to avoid recurrence of drug induced cardiotoxicity which might have been life-threatening. We adopted the arsenic trioxide study protocol, designed by Mathews et al. [10]. Induction therapy was continued with single-agent ATO. She had an uneventful course and achieved hematological complete remission (HCR) after 38 days. Bone marrow examination was repeated after an interval of four weeks and HCR was confirmed. Cytogenetic study carried out on the cells from the bone marrow aspirate showed normal karyotype.

**Figure 3:**
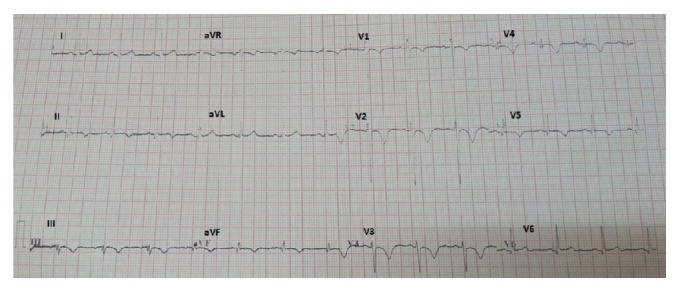
Electrocardiogram obtained 1 month after the acute coronary syndrome. It shows normalization of T wave in V6, I, and aVL. There is inversion of T wave and q wave in leads II, III, and aVF.

**Figure 4:**
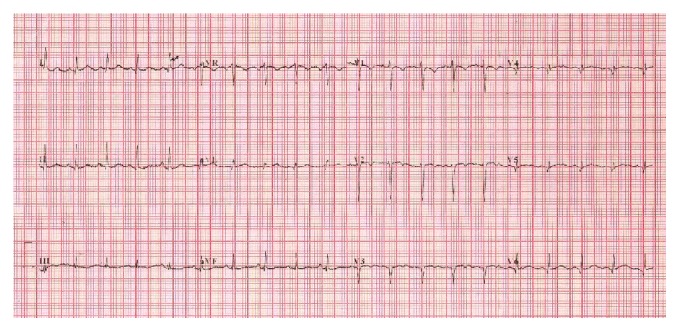
Electrocardiogram obtained 2 months after the acute coronary syndrome. It shows normalization of T wave in leads I, aVL, II, III, aVF, and V4–V6.

**Figure 5:**
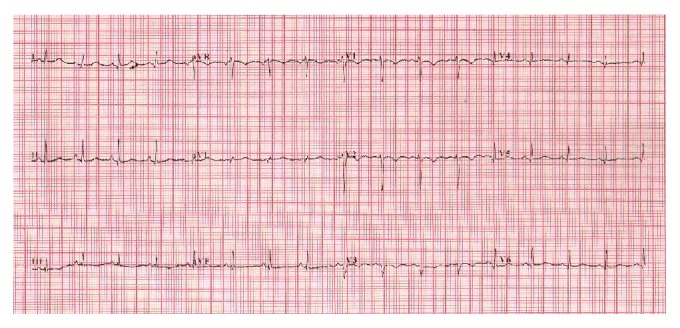
Electrocardiogram obtained 4 months after the acute coronary syndrome. It shows normalization of R wave progression.

**Figure 6:**
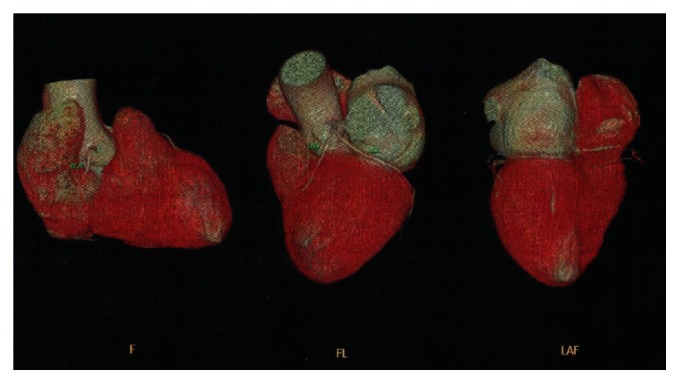
CT coronary angiography. There is no significant stenosis in the coronary arteries.

**Figure 7:**
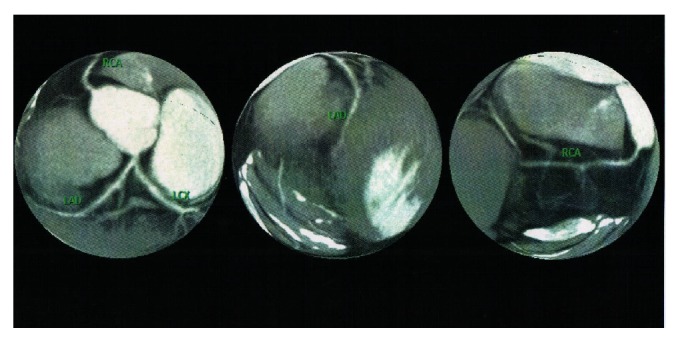
CT coronary angiography. There is no significant stenosis in the coronary arteries.

Serial electrocardiography showed normalization of negative T wave in leads V4, V5, and V6 (Figures 3, 4, and 5). 64-slice computed tomography angiography was performed forty days after the acute coronary syndrome, following normalization of the hematological parameters. There was no significant coronary stenosis (Figures 6 and 7).

She is currently undergoing consolidation therapy with ATO and is followed up by the cardiologist regularly. She has not had further episodes of myocardial ischemia. MUGA study carried out four months after the episode of acute myocardial ischemia showed no regional wall motion abnormalities. LVEF was 52%.

## 3. Discussion

The protocols for the treatment of APL have evolved to include various combinations of ATRA and chemotherapy. This has reduced the early mortality and increased remission rates [11, 12]. ATRA is not without side effects, albeit generally mild. This is the case report of a lady who had an acute coronary syndrome as an adverse effect of ATRA in APL.

Extensive review of literature revealed only few cases describing ATRA induced myocardial toxicity. Latagliata et al. studied the role of ATRA in newly diagnosed APL in 20 patients aged > 60 years. Six episodes of acute myocardial ischemia were observed in six patients. Four patients had myocardial infarction (MI) that was fatal in two and two had unstable angina. Four patients had previous cardiovascular disease, in particular, arterial hypertension, atrial fibrillation, occlusive arterial disease, and myocardial infarction [4]. The case reports of MI, coronary vasospasm, and myocardial stunning associated with ATRA have been summarized in Table 3.

To our knowledge, ours was the youngest patient to be diagnosed with ATRA induced acute myocardial ischemia. She had neither comorbidities nor APL-DS.

During the episode, her corrected QT interval was 440 milliseconds. The normal value for adult female is <450 milliseconds [13]. She had no further episodes of myocardial ischemia after resuming therapy with single-agent ATO. So, ATO was not considered the causative agent.

ATRA is a vitamin A derivative used in the treatment of APL. The APL-DS is seen in approximately 26% of cases [14], typically within 21 days of treatment. It is characterized by fever, dyspnea, hypotension, and pleural and pericardial effusions [15]. Other manifestations include respiratory distress, pulmonary infiltrates, pulmonary edema, and acute renal failure [8]. Approximately 17% of patients had significant decline in LVEF [8]. There are reports of coronary vasospasm and myocardial stunning in the setting of APL-DS [6, 7]. Our patient did not have APL-DS. Dombret et al. have postulated a thrombophilic effect of ATRA characterized by prolonged persistence of procoagulant activity [16]. This is the most likely mechanism of acute coronary syndrome in our patient, although it is difficult to assess in the clinical setting. She was not rechallenged with ATRA as we were concerned about life-threatening cardiotoxicity.

**Figure 8:**
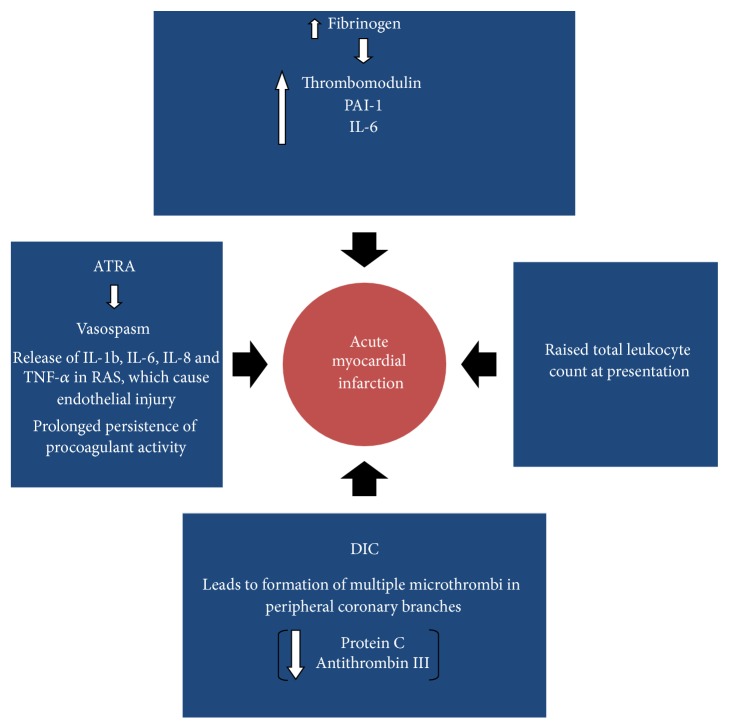
The possible mechanisms of acute coronary syndrome in the setting of APML.

We reviewed all possible mechanisms of acute coronary syndrome in the setting of APML. This has been summarized in a diagram in Figure 8.

*(1) ATRA Induced.* ATRA can exacerbate the procoagulant state of APML [16]. The risk of thromboembolism may be as high as 5%. There is evidence that ATRA reduces the expression of markers of activation of coagulation more rapidly and completely than those of activation of fibrinolysis. However, the exact mechanism is unclear [1, 17]. It can cause coronary vasospasm [6, 7].

*(2) APL Differentiation Syndrome.* In APL-DS, the promyelocytes release IL-1b, IL-6, IL-8, and TNF-*α* resulting in endothelial damage. Tissue infiltration by promyelocytes may also play a role [18]. Our patient did not have APL-DS.

*(3) Disseminated Intravascular Coagulation.* DIC causes multiple microthrombi in peripheral branches of the coronary arteries which can result in severe cardiac dysfunction [6, 19, 20]. Patients in DIC have lower levels of anticoagulants such as protein C and antithrombin III [21–23]. Though our patient was in DIC at the time of diagnosis, her PT and aPTT had returned to normal while she developed the acute coronary syndrome (Table 1).

*(4) Elevated Fibrinogen Levels.* Patients with high fibrinogen levels (>200 mg/dL) have less activation of secondary fibrinolysis [24] leading to the formation of microthrombi. It also contributes to increase in clot stiffness, fiber density, platelet binding, and blood viscosity [25]. Our patient had elevated fibrinogen levels (500 mg/dL).

*(5) In APML.* The risk of thrombosis has been correlated with raised white blood cell count, presence of bcr3 isoform subtype of PML/RAR*α* and fms-like tyrosine kinase 3 internal tandem duplication (FLT3 ITD) mutations, and expression of CD2 and CD15 [17]. Our patient had elevated total count at diagnosis, which continued to increase at the time of the acute coronary syndrome, as shown in Table 1. She did not have the other factors mentioned above.

We treated her in accordance with the arsenic trioxide study protocol by Mathews et al. [10]. This protocol incorporates single-agent ATO, 10 mg/day, administered as an intravenous infusion once daily. It consists of three phases, induction, consolidation, and maintenance. Induction is continued till the patient is in complete remission, for a maximum duration of 75 days, followed by a period of rest of four weeks. During consolidation, ATO is given for four weeks. If the patient is in complete remission, maintenance therapy with ATO is given for ten days every month for six months. However, we planned to consolidate with two courses of ATO with four weeks rest in between. Hemoglobin, total and differential counts, platelet count, renal and liver function tests, and serum potassium, magnesium, and calcium are monitored weekly. Electrocardiogram is repeated weekly to look for prolongation of corrected QT interval (QTc). While reviewing literature we found multiple mechanisms of ATRA induced cardiotoxicity. So, she was not rechallenged with ATRA, even in conjunction with an anticoagulant as we wanted to avoid recurrence of drug induced cardiotoxicity which may be life-threatening. We were concerned about further interruptions and delay in treatment of APML, should the cardiotoxicity recur with a rechallenge, which may jeopardize the treatment outcome.

## 4. Conclusion

Acute coronary syndrome is an infrequent, yet important adverse effect of ATRA. Early diagnosis is life-saving. Single-agent ATO is a valid treatment option for these patients who cannot avail the benefits of ATRA and anthracyclines.

## Figures and Tables

**Table 1:** Coagulation profile during the course of treatment.

	Day 1	Day 9	Day 10	Day 16	Day 25
Fibrinogen (180–350 mg/dL)	247 mg/dL	500 mg/dL	Patient had acute myocardial infarction	210 mg/dL	190 mg/dL
PT (INR)	Prolonged (no clot for >120 seconds)	Normal (1.4)		Normal (1.4)	Normal (1.2)
aPTT (control = 32 seconds)	Prolonged (>50 seconds)	Normal (29 seconds)		Normal (29.2 seconds)	Normal (29.2 seconds)
Platelet count (1,50,000–4,00,000/mm^3^)	18,000/mm^3^	56,000/mm^3^		2,52,000/mm^3^	4,96,000/mm^3^
Interpretation	DIC	Thrombocytopenia, not in DIC		Normal	Normal
Total count (4,000–11,000/mm^3^)	10,040/mm^3^	10,400/mm^3^		2,800/mm^3^	3,900/mm^3^

**Table 2:** The Naranjo Adverse Drug Reaction Probability Scale [9].

		Yes	No	Do not know	Score
1	Are there previous conclusive reports on this reaction?	+1	0	0	+1
2	Did the adverse event occur after the suspected drug was administered?	+2	−1	0	+2
3	Did the adverse reaction improve when the drug was discontinued?	+1	0	0	+1
4	Did the adverse reaction reappear when the drug was readministered?	+2	−1	0	0
5	Are there alternative causes (other than the drug) that could have on their own caused the reaction?	−1	+2	0	+2
6	Did the reaction reappear when a placebo was given?	−1	+1	0	—
7	Was the drug detected in the blood (or other fluids) in a concentration known to be toxic?	+1	0	0	—
8	Was the reaction more severe when the dose was increased or less severe when the dose was decreased?	+1	0	0	—
9	Did the patient have a similar reaction to the same or similar drugs in any previous exposure?	+1	0	0	—
10	Was the adverse event confirmed by any objective evidence?	+1	0	0	+1

Total score					**7**
This is a probable adverse effect of ATRA					

She was not rechallenged with ATRA in order to avoid recurrence of drug induced cardiotoxicity which might be life-threatening.

**Table 3:** Case reports of acute myocardial ischemia associated with ATRA.

Authors	Age (years)	Gender	Comorbidities	Presence of APL-DS	Type of myocardial ischemia	Treatment	Outcome
Tallman et al. [8]	52	Male	None	Yes	Acute myocardial infarction	ATRA was discontinued.	Patient died.

Miyoshi et al. [5]	71	Male	None	Yes	Acute myocardial infarction (he also had lacunar infarction of right lentiform nucleus)	ATRA was discontinued. Methyl prednisolone and gabexate mesilate were administered.	Patient recovered.

De Santis et al. [6]	76	Female	None	Yes	Myocardial stunning; nonobstructive coronary artery disease	ATRA was discontinued. She was prescribed nitrates and dexamethasone.	Patient recovered. ATRA was restarted. She tolerated treatment well. Echocardiogram done after two months showed normal left ventricular ejection fraction and no segmental hypocontractility.

Maqsood et al. [7]	65	Male	Hypertension, ulcerative colitis	Yes	Coronary vasospasm mimicking ST-elevation myocardial infarction	ATRA was discontinued. Nitroglycerine, beta-blockers, and dexamethasone were given. Dopamine infusion was started as the patient was hypotensive.	Patient recovered.

Our patient	42	Female	None	No	Acute coronary syndrome with left ventricular failure	ATRA was discontinued. She was prescribed metoprolol, ramipril, nitrates, and diuretics.	Patient recovered.

## Abbreviations

ADR: Adverse drug reaction
aPTT: Activated partial thromboplastin time
ATRA: All-*trans*-retinoic acid
APL: Acute promyelocytic leukemia
ATO: Arsenic trioxide
APL-DS: Acute promyelocytic leukemia-differentiation syndrome
CD: Cluster of differentiation
DIC: Disseminated intravascular coagulation
FLT3-ITD mutation: Fms-like tyrosine kinase 3 internal tandem duplication mutation
HCR: Hematological complete remission
HLA: Human leukocyte antigen
IL: Interleukin
LVEF: Left ventricular ejection fraction
MI: Myocardial infarction
MPO: Myeloperoxidase
MUGA study: Multigated acquisition mode study
PML/RAR*α*: Promyelocytic leukemia/retinoic receptor alpha fusion protein
PT (INR): Prothrombin time (international normalized ratio)
QTc: Corrected QT interval
TNF-*α*: Tumor necrosis factor-alpha.
